# Moral Injury and COVID‐Related Stress Among Post‐9/11 Veterans: Examining Longitudinal Associations

**DOI:** 10.1002/smi.70121

**Published:** 2025-11-10

**Authors:** Ryan P. Chesnut, Cameron B. Richardson, Keith R. Aronson, Daniel F. Perkins

**Affiliations:** ^1^ Clearinghouse for Military Family Readiness The Pennsylvania State University University Park Pennsylvania USA; ^2^ Social Science Research Institute The Pennsylvania State University University Park Pennsylvania USA; ^3^ Department of Biobehavioral Health The Pennsylvania State University University Park Pennsylvania USA; ^4^ Department of Agricultural Economics The Pennsylvania State University Sociology and Education University Park Pennsylvania USA

**Keywords:** COVID‐19, mental health, moral injury, potentially morally injurious experiences, structural equation modelling, veterans

## Abstract

Although most U.S. military veterans make a successful transition from military service to civilian life, some may experience a heightened risk for having adverse mental health symptoms related to moral injury (MI), post‐traumatic stress disorder (PTSD), depression, and anxiety. In addition, U.S. veterans may have faced an increased risk for experiencing more pronounced stress across multiple life domains during the COVID‐19 pandemic. Limited research attention has been given to understanding how MI symptoms experienced by post‐9/11 veterans prior to the COVID‐19 pandemic (COVID) were related to their experiences of COVID‐related stress. This study examined the impact of pre‐COVID MI symptoms on veterans' COVID‐related stress in various life domains. Participants included 3180 U.S. veterans who were involved in The Veterans Metric Initiative (TVMI) and the Veterans Engaging in Transition Studies (VETS). TVMI was a longitudinal study that occurred from 2016 to 2019. VETS, which began in 2020, was a continuation of TVMI and included a portion of the TVMI sample. Data from assessments of mental health variables from TVMI Wave 6 (spring 2019), COVID‐related work, financial, social, and health stress from VETS Wave 7 (fall 2020), and relevant covariates from TVMI Wave 1 (fall 2016) were included in structural equation modelling analyses. Results demonstrated that MI predicted COVID‐related work and financial stress, PTSD did not predict any of the four COVID‐related stress domains, and depression and anxiety predicted all four COVID‐related stress factors. The study's findings highlight the nuanced ways in which veterans' psychological experiences can impact their feelings of stress during a global pandemic.

## 1 Introduction

Moral injury (MI) refers to the negative intrapersonal experiences one may have in response to violations of his or her deeply held moral convictions (Litz et al. 2009; Shay 2010). Morally injurious experiences are thought to involve the perception of moral failure by the self or others (Atuel et al. 2021; Litz et al. 2009). This idea of moral failure is typically tied to the notion of transgression (VanderWeele et al. 2025). Thus, self‐directed MI occurs when a person transgresses (i.e., betrays) his or her morals and ethics, while other‐directed MI occurs when someone else's actions or decisions transgress one's morals and ethics (Atuel et al. 2020, 2021). Recently, though, some MI scholars have advanced a definitional framework for MI that allows for the possibility that MI can arise from non‐transgression experiences (e.g., dilemmas, struggles, natural events; VanderWeele et al. 2025). In this broader framework, potentially morally injurious events (PMIEs) could include a wide variety of human experiences such as killing people while in combat, witnessing atrocities of war, being betrayed by trusted others in dangerous situations, lying, violating another's privacy, disregarding another's discomfort or distress, struggling with an addiction, or witnessing an act of nature that challenges one's worldview.

Moral emotions are considered integral to the experience of MI (Farnsworth et al. 2014). Self‐directed MI is expected to produce moral emotions such as guilt, shame, loss of faith, and social withdrawal (Farnsworth et al. 2014; Fleming and Smigelsky 2024; Litz et al. 2009), while other‐directed MI is posited to produce emotional reactions like anger, disgust, and contempt (Farnsworth et al. 2014; Fleming and Smigelsky 2024). When an individual struggles with MI over time, numerous dysregulated cognitive, affective, and behavioural outcomes can manifest, such as mental health symptoms, social isolation, and suicidal ideation (Chesnut et al. 2020; Griffin et al. 2019). It is important to note, with respect to mental health symptoms, that MI, as it is currently understood, is related to and yet distinguishable from posttraumatic stress disorder (PTSD) and internalizing disorders, such as depression and anxiety (Griffin et al. 2019; Williamson et al. 2018).

### 1.1 Post‐9/11 Veterans and Moral Injury

During wartime, service members are likely to face numerous moral situations that place them at risk for experiencing MI (Litz et al. 2009). For example, service members may engage in behaviours (e.g., killing enemy combatants) that are allowed during combat but are criminal actions outside of warfare. Moreover, they may engage in behaviours that are not sanctioned under military rules of engagement and international law, such as killing or harming civilians and engaging in war atrocities. These behaviours have been directly associated with service members experiencing MI (Frankfurt and Frazier 2016) and mental health problems (Maguen et al. 2009) such as PTSD, depression, anxiety, and substance abuse; suicidality (Nichter, Norman, et al. 2021); and other psychosocial problems such as social isolation (Chesnut et al. 2020; Barnes et al. 2019; McEwen et al. 2021; Wisco et al. 2017).

The number of veterans who experience PMIEs or have MI, however, is not known as few epidemiologic studies have been completed. Analysis of data from the second baseline cohort of the National Health and Resilience in Veterans Study (NHRVS) collected in 2013 found that, of the 564 veterans who reported having been in combat, 25.5% endorsed (i.e., selected ‘moderately agree’ or ‘strongly agree’) at least one morally injurious transgression by others item on the Moral Injury Events Scale (MIES); 25.5% endorsed at least one MIES betrayal item; 10.8% endorsed at least one MIES transgression by self item; and 41.8% endorsed any MIES item (Wisco et al. 2017). Analysis of more recent data from the 2019‐2020 NHRVS (Nichter, Norman, et al. 2021) that included 1321 veterans with combat or warzone exposure found similar rates of endorsement (i.e., transgressions by others: 23.1%; betrayal: 24.5%; transgression by self: 11.2%; and any MIES item: 36.8%). Though Service members, clearly, experience MI and PMIEs, the study of MI remains underdeveloped, and calls for research examining MI as a unique psychological construct persist (Griffin et al. 2019; Litz 2025).

### 1.2 The COVID‐19 Pandemic as a Risk to Veteran Well‐Being

A growing number of studies have found that the COVID‐19 (COVID) pandemic contributed to high levels of stress for individuals across various life domains (Barzilay et al. 2020; Gallagher et al. 2020). In the United States (U.S.), approximately 25% of adults experienced significant stress due to the COVID pandemic (Cooke et al. 2020). In addition, the COVID pandemic subjected different portions of the U.S. population to PMIEs (Borges et al. 2020; Shale 2020; Williamson et al. 2020). While MI could theoretically be relevant to anyone who experienced PMIEs during the COVID pandemic (Shale 2020), given the nature of the pandemic, MI research predominately focused on healthcare workers and first responders (Khan et al. 2021; M. A. Wilson et al. 2024). In particular, healthcare workers were at risk of experiencing PMIEs that could produce MI‐specific symptoms along with other psychological symptoms (e.g., depression). For example, ethical concerns experienced by healthcare workers during the COVID pandemic (e.g., working with limited resources, limiting or forgoing interventions for patients, not being able to advocate for patients due to fear of negative consequences) were significantly associated with experiencing MI symptoms (Rushton et al. 2022). A recent scoping review found that prevalence estimates of MI among healthcare workers during the COVID pandemic ranged from 25% to 41% (Riedel et al. 2022). Moreover, studies comparing MI/PMIE prevalence rates between combat veterans and healthcare workers have found generally comparable estimates (Maguen et al. 2025; Nieuwsma et al. 2022). For instance, Nieuwsma et al. (2022) found that 46.1% of combat veterans and 50.7% of healthcare workers reported experiencing an other‐directed PMIE. Further, they found that PMIEs were associated with greater depressive symptomology and lower quality of life in healthcare workers during the COVID pandemic, which corresponds with other research focused on the adverse impact of MI on the mental health of healthcare workers (Coimbra et al. 2024). Unfortunately, prior work has not examined the association between MI and veteran well‐being during the COVID pandemic, and moreover, has not examined how veterans' pre‐COVID MI symptoms may have impacted their well‐being during the pandemic.

Veterans are an important subgroup of the larger U.S. population that may have been at heightened risk for experiencing poor outcomes during the COVID pandemic (Hill et al. 2023). There are several reasons for veterans' increased susceptibility. First, veterans are considered an at‐risk or vulnerable population (Olenick et al. 2015). Compared to civilians, veterans have higher rates of physical disability and medically complex health issues (Betancourt et al. 2023), are more likely to have been diagnosed with PTSD (Lehavot et al. 2018), report more indicators of social isolation (Vespa 2023), are at increased risk for suicide (Morral et al. 2023), and are less likely to seek mental health treatment due to perceptions of stigma (Clement et al. 2015). Second, veterans report greater exposure to adverse childhood experiences (ACEs) than civilians (Blosnich et al. 2021), and veterans may have experienced military‐specific trauma, such as combat‐related trauma and/or military sexual trauma (Schafer et al. 2022). Prior exposure to trauma is associated with poorer health and well‐being outcomes for veterans who are exposed to an additional stressor (Davis et al. 2023). Third, after completing active duty service, every veteran must make a military‐to‐civilian transition (MCT). MCTs require veterans to prepare to leave the military, re‐orient to civilian life, adapt to civilian life, and ideally thrive over time (Blackburn 2017; Robinson et al. 2017). Veterans must also contend with losing their military identity, regimentation, and close friends and colleagues (Pedlar et al. 2019). A considerable minority of veterans struggle with this process (Karre et al. 2024; Park et al. 2021), and more than one‐quarter report the transition as somewhat or very difficult (Morin 2011).

Studies examining veterans' health and well‐being during the COVID pandemic indicate that, overall, veterans were resilient (Fischer, Nichter, et al. 2023; Hill et al. 2023; Kalvesmaki et al. 2022; McGuire et al. 2023; Na et al. 2022; Pedersen et al. 2023; Spiller et al. 2023). That said, several studies noted increased mental health symptomology (Betancourt et al. 2023; Fischer, Na, et al. 2023; Hill et al. 2023; S. Li et al. 2023; Spiller et al. 2023). Further, a small, but meaningful, proportion of veterans were found to develop new‐onset suicidal ideation and planning (Fischer, Na, et al. 2023; Nichter, Hill, et al. 2021). A few studies reported increased perceptions of social isolation and loneliness (Kalvesmaki et al. 2022; S. Li et al. 2023; Purcell et al. 2021) with one study that used 2019‐2020 NHRVS data finding 5.4% of the veteran sample reported increased loneliness and 10.8% reported persistent loneliness during the COVID pandemic (Na et al. 2022). Pre‐pandemic psychiatric symptoms have consistently been linked to veterans' behavioural, mental, and physical health functioning during the COVID pandemic (Fein‐Schaffer et al. 2022; Fischer, Na, et al. 2023; S. Li et al. 2023; Na et al. 2022; Nichter, Hill, et al. 2021). However, existing research examining associations among pre‐COVID mental health and veteran health and well‐being during the pandemic have not included pre‐COVID levels of MI. Further, while the extant literature has focused on important health and well‐being outcomes for veterans, veterans' experiences of COVID‐related stress in various life domains remain understudied, even though veterans have reported experiencing distress in multiple life domains due to the COVID pandemic (Aronson et al. 2024; Kalvesmaki et al. 2022).

### 1.3 The Current Study

Given the importance of studying the COVID pandemic experiences of vulnerable groups (Holmes et al. 2020; O'Connor et al. 2020), like veterans, and the significance of MI for the veteran population (Farnsworth et al. 2014; Frankfurt and Frazier 2016; Maguen et al. 2023; Nichter, Norman, et al. 2021; Wisco et al. 2017; Zerach et al. 2023), the current study sought to examine the degree to which the level of pre‐COVID MI was associated with reports of stress across various life domains, among a large sample of post‐9/11 veterans, during the COVID pandemic.

A strength of the current study was its longitudinal design and inclusion of PTSD, anxiety, and depression symptoms as covariates to test whether MI accounts for unique variance in the presence of other mental health variables known to impact veteran functioning. Examining MI in combination with PTSD and internalizing symptoms has been identified as an important MI research focus because it will help to enhance the conceptual clarity of the MI construct (Griffin et al. 2019; Litz 2025). It was hypothesized that pre‐COVID levels of MI would be associated with higher levels of stress during the COVID pandemic.

## 2 Method

### 2.1 Participants and Procedures

The data for the present study come from Waves 1 and 6 of The Veterans Metric Initiative (TVMI) and Wave 7 of the Veterans Engaging in Transition Studies (VETS), which was a continuation and expansion of the TVMI study. TVMI was a longitudinal study of post‐9/11 veterans' transition from military to civilian life. Detailed information about the TVMI study, including study objectives, design details, and participant demographics, has been published elsewhere (Vogt et al. 2018). In summary, 48,965 veterans who had separated or deactivated from active duty status between August and November 2016, had at least 180 days of military service in an active duty component service branch or deactivated from active duty status after serving at least 180 days in a reserve component, and had a valid U.S. mailing address were identified via the Veterans Affairs/Department of Defence Identity Repository (VADIR) and were invited to participate in the study. From this population, 9566 veterans completed the TVMI Wave 1 survey in fall 2016. Participants received additional surveys every 6 months over a 2.5‐year period for a total of six waves of data collection. The sixth and final TVMI survey was administered in spring 2019. About 55% of the Wave 1 sample (*n* = 5258) completed the Wave 6 survey. A $5 cash incentive was sent to participants in advance of administering the Wave 1 survey to boost response rates. Participants received a $20 e‐gift card for completing the Wave 1 survey, and this incentive increased by $5 at each wave until the amount reached $50. Ethical approval was obtained from IFC International Inc., and consent was implied for those completing the Wave 1 survey.

The average age of the veterans at Wave 6 was 36.40 years (SD = 9.42), the majority were male (82%), White (68%), enlisted (76%), and 70% had experienced at least one combat deployment. Service component representation was greatest for the Army (32%) followed by the Air Force (20%), Navy (19%), Marine Corps (17%), and National Guard or Reserves (12%).

Over one‐third of the original TVMI sample (36.8%; *n* = 3516) expressed interest in continued research opportunities and were invited to participate in VETS. Of this group, 3180 veterans completed the Wave 7 survey, which was administered in fall 2020. As the VETS Wave 7 survey occurred during the COVID pandemic and lockdowns in the U.S., the survey included a series of items that asked about COVID‐specific stress. Ethical approval for VETS was obtained from the Institutional Review Board of the Pennsylvania State University. Participants provided consent prior to starting the Wave 7 survey, and they were compensated with a $50 e‐gift card for completing the survey. The average age of the respondents was 37.91 years (SD = 9.41), and the majority were male (81%), White (70%), enlisted (75%), and had experienced at least one combat deployment (69%). Service component representation was greatest for the Army (32%) followed by the Air Force (20%), Navy (20%), Marine Corps (16%), and National Guard or Reserves (12%). Participants were included in the analyses, described below in the analysis plan section, if they completed the Wave 7 survey (*n* = 3180).

### 2.2 Measures

#### 2.2.1 Moral Injury—Moral Injury Symptoms Scale—Military ‐ Short Form

The Moral Injury Symptoms Scale—Military—Short Form (MISS‐M‐SF; Koenig et al. 2018a) was administered at Wave 6. The MISS‐M‐SF is a 10‐item instrument developed to measure the psychological (e.g., shame, guilt) and spiritual (e.g., loss of faith) symptoms associated with MI (Koenig and Al‐Zaben 2020). The full MISS is comprised of 45 items, many of which were taken from existing measures (e.g., Moral Injury Events Scale, Combat Guilt Scale, Rosenberg Self‐Esteem Scale), across 10 subscales (Koenig et al. 2018b). The item with the highest factor loading from each subscale was selected for inclusion on the MISS‐M‐SF. For the first nine items, respondents indicate their level of agreement with each statement on a 10‐point Likert scale (1 = *strongly disagree* to 10 = *strongly agree*). For item 10, participants indicate the extent to which their religious faith has improved or deteriorated since joining the military on a 10‐point Likert scale (1 = *strengthened a lot* to 10 = *weakened a lot*). Items 1–4 and 8–9 are negatively worded, and items 5–7 and 10 are positively worded. The inclusion of items with differing valence complicates the dimensionality of the scale by introducing extraneous method factors. Results of a bifactor model analysis showed that the MISS‐M‐SF can be considered essentially unidimensional, thereby relieving investigators from the burden of having to model the extraneous method factors, which can complicate latent variable analyses (Chesnut et al. 2022). That said, the same study also demonstrated that a reduced set of four items (i.e., 1, 4, 8, and 9), with comparable psychometric properties to the 10‐item scale, could be used to efficiently model MI symptomology (MISS‐M‐SF4). Thus, for the sake of model parsimony, the MISS‐M‐SF4 items were used to model MI symptomology as a latent variable (McDonald's omega = 0.75). The MISS‐M‐SF and MISS‐M‐SF4 have been previously validated with veteran samples (Chesnut et al. 2022; Koenig et al. 2018a).

#### 2.2.2 PTSD—Abbreviated PTSD Checklist for DSM‐5

At Wave 6, PTSD symptomology was assessed with the abbreviated 4‐item PTSD Checklist for DSM‐5 (PCL‐5; Price et al. 2016). The 4‐item abbreviated PCL‐5 assesses the PTSD symptom clusters of intrusion (i.e., ‘Repeated, disturbing, and unwanted memories of the stressful experience(s)’), avoidance (e.g., ‘Avoiding external reminders of the stressful experience(s)’), negative alterations in cognition and mood (e.g., ‘Having strong negative beliefs about yourself, other people, or the world’), and alterations in arousal and reactivity (e.g., ‘Feeling jumpy or easily startled’) over the past month using a 5‐point Likert‐type scale (0 = *Not at all* to 4 = *Extremely*). The items were only administered to those veterans who indicated at Wave 6, or at any previous wave, that they had experienced an unusually or especially frightening, horrible, or traumatic event in their lifetime (e.g., serious accident or fire, warzone exposure, physical assault or abuse). Veterans who indicated they had not experienced such an event in their lifetime at Wave 6 and all previous waves were given a score of 0 for each item. PTSD symptomology was treated as a latent variable (McDonald's omega = 0.91). The 4‐item abbreviated PCL‐5 was previously validated in a veteran sample (Price et al. 2016).

#### 2.2.3 Depression and Anxiety—Patient Health Questionnaire‐4

The 4‐item Patient Health Questionnaire (PHQ‐4; Kroenke et al. 2009) was administered at Wave 6. The PHQ‐4 assesses the two core symptoms of depression (e.g., ‘Little interest or pleasure in doing things’) and anxiety (e.g., ‘Feeling nervous, anxious, or on edge’) over the previous 2 weeks using a 4‐point Likert‐type scale (0 = *Not at all* to 3 = *Nearly every day*). Depression and anxiety were modelled as latent variables (McDonald's omega for depression = 0.84; McDonald's omega for anxiety = 0.86). The PHQ‐4 has been previously validated in a veteran sample (Kroenke et al. 2019).

#### 2.2.4 COVID‐Related Stress

At Wave 7, a series of items, developed by the research team, was administered to assess participants' experiences of social, work, financial, and health stress related to the COVID‐19 pandemic. The items that assessed social, work, and financial stress were proceeded by the stem ‘How stressful have these aspects of the COVID‐19 pandemic been for you?’ Social stress was comprised of 3 items (e.g., ‘…being socially isolated’), work stress consisted of 4 items (e.g., ‘…stability of job’), and financial stress included 2 items (e.g., ‘…paying for essential items’). Health stress consisted of 2 items that asked participants about how stressful the COVID‐19 pandemic had been on them physically and mentally/emotionally. All items were rated on a 5‐point Likert‐type scale (0 = *Not at all* to 4 = *Extremely*), and each domain was treated as a latent variable (McDonald's omega for social stress = 0.87; McDonald's omega for work stress = 0.75; McDonald's omega for financial stress = 0.83; and McDonald's omega for health stress = 0.78).

#### 2.2.5 Covariates

A series of covariates assessed at Wave 1 were included in the structural equation modelling (SEM) analyses due to their potential relevance to the study's substantive variables. The covariates included biological sex (0 = *female* to 1 = *male*), race/ethnicity (series of dichotomous variables constituting *White* [reference group], *Black*, *Hispanic*, *Asian/Pacific Islander/Hawaiian*, *Multiracial/ethnic*, and *Other race/ethnicity*), military paygrade (1 = *E1‐E4* to 6 = *O4‐O10*), military service component (series of dichotomous variables constituting *Army* [reference group], *Navy*, *Marine Corps*, *Air Force*, and *National Guard/Reserve*), military occupation (series of dichotomous variables constituting *Combat Arms* [reference group], *Combat* *Support*, and *Service* *Support*), and combat exposure (9 items adapted from the DDRI‐2, 0 = *Never* to 3 = *Many Times*; McDonald's omega = 0.96; previously validated in a veteran sample [National Academies of Sciences et al. 2018]). All covariates were treated as observed variables in the SEM analyses except for combat exposure, which was modelled as a latent variable.

### 2.3 Analysis Plan

The lavaan (v0.6‐19; Rosseel 2012) and lavaan.mi (v0.1‐0.0030; Jorgensen 2024) R packages were used to perform SEM analyses of the data. Latent factor variances were fixed at 1 for model identification purposes. The weighted least squares mean and variance adjusted (WLSMV) estimator with theta parameterization was employed due to the ordinal nature of most of the study variables (DiStefano and Morgan 2014). MI was considered a continuous variable since the MISS‐M‐SF4 uses a 10‐point scale (Rhemtulla et al. 2012), and the WLSMV estimator accommodates latent variable models with ordinal and continuous indicators (Muthen 1984).

**FIGURE 1:**
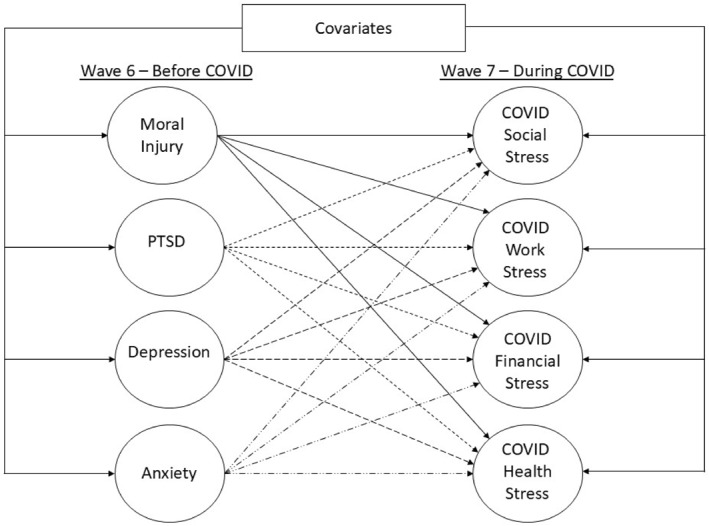
Hypothesized structural model. PTSD = post‐traumatic stress disorder. Latent variable indicators, individual covariates, and latent variable residual correlations are not shown for presentational clarity. Individual covariates include gender, race/ethnicity, military paygrade, military service component, military occupation, and combat exposure.

A two‐phase modelling approach was used that involved specifying a measurement model prior to specifying the hypothesized structural model shown in Figure 1. For the structural model, all substantive study variables were regressed on the demographic and military covariates to obtain estimates of the effects of MI, PTSD, depression, and anxiety symptoms on COVID‐related stress that fully account for the effects of the covariates. The false discovery rate was used to control Type I error inflation due to testing multiple parameters (Cribbie 2007). The Wave 7 survey weight was applied to adjust the sample based on gender, service branch, and paygrade to more closely align with the sampling frame and account for study attrition.

For the measurement and structural models, the standardized root mean square residual (SRMR; ≤ 0.08) was the primary index used to gauge the adequacy of data‐model fit due to the SRMR's robustness to ordinal data estimation methods (Shi and Maydeu‐Olivares 2020; Shi et al. 2020). The root mean square error of approximation (RMSEA; ≤ 0.06), comparative fit index (CFI; ≥ 0.95), and chi‐square (*χ* ^*2*^) are also reported given their historical significance to SEM methodology (Hu and Bentler 1999), but each was given less weight than the SRMR in the model‐evaluation process because of known issues with sensitivity to sample size (i.e., *χ* ^*2*^) or ordinal data estimation methods (i.e., RMSEA, CFI; Shi, Lee, et al. 2020). For the *χ* ^*2*^, CFI, and RMSEA statistics, scaled values calculated using the WLSMV estimator are reported.

Missing data at the item level for the COVID‐related stress variables and the covariates were minimal (0.0%–0.9%); however, item‐level missingness for the mental health variables was more substantial (18.5%–20.0%). The higher percentage of missingness on the mental health variables was predominately due to approximately 600 veterans who did not respond to the Wave 6 survey but did respond to the Wave 7 survey. Little's Missing Completely at Random test was not statistically significant (*χ* ^*2*^[1606] = 1525.48, *p* = 0.924), and, as such, the data were assumed to be missing in a random fashion. Multiple imputation, which involved creating 20 multiply imputed data sets (Graham et al. 2007) with the Amelia II package (Honaker et al. 2011), was employed because this technique has been found to perform well when the missingness mechanism is random even when item‐level missingness is high for both ordinal (< 50%; Shi et al. 2020) and continuous data (50% or more; Enders 2010). Rubin's rules (Rubin 1987) were used to pool parameter estimates and standard errors, and the D2 method (K. H. Li et al. 1991) was used to pool robust model fit indices. Two sensitivity analyses were conducted for the structural model: (a) WLSMV estimation with listwise deletion, and (b) a robust maximum likelihood estimator (MLR) with full information maximum likelihood (FIML) for missing data. No power analyses were performed, a priori or retrospectively, for the SEM analyses conducted.

## 3 Results

### 3.1 Measurement Model

The measurement model contained only the latent variables to be included in the structural model (see Table 1 for the latent variable correlation matrix). The model showed good fit to the data: *χ* ^*2*^ (459) = 1663.59, *p* < 0.001, CFI = 0.979, RMSEA = 0.029 (90% CI [0.027, 0.030]), and SRMR = 0.038. Two issues, however, were detected with this model. First, the correlation between depression and anxiety was excessively high (*r* = 0.89). Such a high correlation between latent factors (*r* > 0.85) reflects poor discriminate validity (Brown 2006) and could result in issues with estimating regression weights and standard errors in the structural model due to multicollinearity. Second, in inspecting the standardized residual matrix to see if there were any large pairwise discrepancies with values noticeably greater than 0.10 in absolute magnitude (Kline 2023), a large discrepancy (0.27) between the observed and implied correlation for items 2 and 3 of the COVID‐related work stress latent variable was found. Both items focus on travel. Item 2 asks about public transportation; item 3 covers work‐related travel.

**TABLE 1:** Latent variable correlation matrix.

Variable	1	2	3	4	5	6	7	8	9
1. Moral injury	—								
2. PTSD	0.65^***^	—							
3. Depression	0.67^***^	0.81^***^	—						
4. Anxiety	0.62^***^	0.82^***^	0.89^***^	—					
5. Combat exposure	0.11^***^	0.33^***^	0.16^***^	0.15^***^	—				
6. COVID social stress	0.13^***^	0.17^***^	0.20^***^	0.23^***^	0.002	—			
7. COVID work stress	0.20^***^	0.18^***^	0.20^***^	0.22^***^	0.03	0.40^***^	—		
8. COVID financial stress	0.33^***^	0.36^***^	0.38^***^	0.36^***^	−0.002	0.36^***^	0.41^***^	—	
9. COVID health stress	0.27^***^	0.34^***^	0.34^***^	0.39^***^	−0.001	0.64^***^	0.63^***^	0.51^***^	—

*Note:* Combat exposure was included in the measurement model because it was treated as a latent variable.

Abbreviation: PTSD = post‐traumatic stress disorder symptoms.

*p < 0.05.

**p < 0.01.

^***^ *p* < 0.001.

The measurement model was re‐specified with depression and anxiety modelled as a single latent variable, and the residuals of the two COVID‐related work stress items were allowed to covary. This respecified model demonstrated good fit to the data with all fit indices showing slight improvement over the originally specified measurement model: *χ* ^*2*^ (466) = 1421.12, *p* < 0.001, CFI = 0.984, RMSEA = 0.025 (90% CI [0.024, 0.027]), and SRMR = 0.036. This respecified measurement model was retained for the structural model analysis.

### 3.2 Structural Model

**FIGURE 2:**
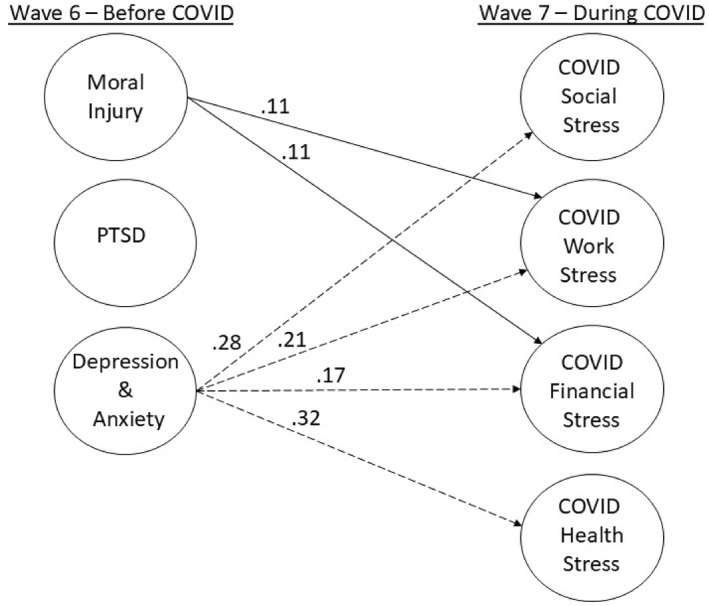
Structural model standardized regression coefficient results. PTSD = post‐traumatic stress disorder. Latent variable indicators, covariates, latent and manifest variable residual correlations, and non‐significant paths are not shown for presentational clarity.

The structural model, which was the respecified measurement model extended to include observed demographic and military covariates, demonstrated a good fit to the data based on the SRMR (0.032) and RMSEA (0.040 [90% CI {0.039, 0.041}]). The *χ* ^*2*^and CFI statistics were not as favourable: *χ* ^*2*^(804) = 4814.68, *p* < 0.001, CFI = 0.908. As described previously, the SRMR was used as the primary index of data‐model fit, and, as such, the structural model was retained as specified. Figure 2 displays the standardized regression coefficients for the paths from the mental health predictors to the COVID stress outcomes that were statistically significant. Demographic and military covariates and nonsignificant pathways are not included in the figure for ease of presentation. Table 2 contains more detailed model‐parameter information.

**TABLE 2:** Structural model regression results.

Mental health predictors & covariates	COVID social stress				COVID work stress				COVID financial stress				COVID health stress			
	*B*	SE	*p*	*β*	*B*	SE	*p*	*β*	*B*	SE	*p*	*β*	*B*	SE	*p*	*β*
Moral injury	−0.03	0.03	0.68	−0.03	**0.11**	**0.04**	**0.01**	**0.11**	**0.12**	**0.04**	**0.009**	**0.11**	0.02	0.04	0.69	0.02
PTSD	−0.04	0.06	0.68	−0.04	−0.06	0.07	0.58	−0.06	0.12	0.07	0.25	0.12	0.05	0.07	0.56	0.05
Depression & anxiety	**0.28**	**0.06**	**< 0.001**	**0.28**	**0.21**	**0.07**	**0.006**	**0.21**	**0.18**	**0.06**	**0.027**	**0.17**	**0.33**	**0.07**	**< 0.001**	**0.32**
Combat exposure	0.002	0.03	0.95	0.002	0.01	0.04	0.85	0.01	−0.04	0.04	0.71	−0.03	−0.03	0.03	0.46	−0.03
Gender	**−0.36**	**0.06**	**< 0.001**	**−0.13**	0.03	0.07	0.85	0.01	−0.03	0.07	0.87	−0.01	**−0.39**	**0.07**	**< 0.001**	**−0.13**
Paygrade	**0.08**	**0.02**	**< 0.001**	**0.12**	**0.05**	**0.02**	**0.006**	**0.07**	**−0.16**	**0.02**	**< 0.001**	**−0.22**	**0.06**	**0.02**	**0.001**	**0.08**
Air force	**0.26**	**0.07**	**0.001**	**0.08**	**0.16**	**0.07**	**0.045**	**0.05**	0.08	0.07	0.69	0.02	0.08	0.07	0.38	0.03
Marines	0.10	0.07	0.31	0.04	−0.06	0.08	0.58	−0.02	0.06	0.08	0.71	0.02	−0.13	0.07	0.12	−0.04
Navy	0.13	0.07	0.15	0.05	−0.01	0.08	0.85	−0.01	0.05	0.08	0.81	0.02	−0.004	0.07	0.95	−0.002
NGR	0.09	0.08	0.40	0.04	**0.20**	**0.08**	**0.029**	**0.08**	0.01	0.08	0.92	0.003	−0.02	0.08	0.84	−0.01
Black	−0.06	0.08	0.68	−0.01	**0.30**	**0.09**	**0.005**	**0.07**	0.20	0.09	0.15	0.05	**0.33**	**0.09**	**0.001**	**0.08**
Hispanic	−0.02	0.07	0.88	−0.01	**0.30**	**0.07**	**0.001**	**0.10**	0.11	0.07	0.40	0.03	**0.30**	**0.07**	**< 0.001**	**0.09**
APINH	**0.38**	**0.15**	**0.044**	**0.06**	**0.54**	**0.16**	**0.005**	**0.08**	0.07	0.16	0.87	0.01	**0.48**	**0.14**	**0.002**	**0.07**
Multiracial/ethnic	0.02	0.10	0.89	0.004	**0.37**	**0.11**	**0.005**	**0.08**	0.04	0.11	0.87	0.01	0.23	0.11	0.07	0.05
Other race/ethnicity	−0.47	0.29	0.24	−0.03	−0.06	0.28	0.85	−0.01	0.03	0.25	0.92	0.002	−0.23	0.28	0.55	−0.02
Service support	0.05	0.07	0.68	0.02	0.02	0.07	0.85	0.01	−0.06	0.07	0.71	−0.03	**0.21**	**0.07**	**0.005**	**0.09**
Combat support	0.03	0.06	0.70	0.02	0.14	0.07	0.07	0.07	−0.01	0.07	0.92	−0.01	**0.20**	**0.07**	**0.005**	**0.09**

Covariates	Moral injury				PTSD				Depression & anxiety			
	*B*	SE	*p*	*β*	*B*	SE	*p*	*β*	*B*	SE	*p*	*β*
Combat exposure	**0.18**	**0.03**	**< 0.001**	**0.17**	**0.44**	**0.03**	**< 0.001**	**0.39**	**0.25**	**0.03**	**< 0.001**	**0.23**
Gender	**−0.19**	**0.06**	**0.004**	**−0.07**	**−0.34**	**0.07**	**< 0.001**	**−0.11**	**−0.30**	**0.07**	**< 0.001**	**−0.10**
Paygrade	**−0.08**	**0.02**	**< 0.001**	**−0.11**	**−0.06**	**0.02**	**0.002**	**−0.08**	**−0.09**	**0.02**	**< 0.001**	**−0.13**
Air force	**−0.16**	**0.07**	**0.031**	**−0.05**	**−0.38**	**0.08**	**< 0.001**	**−0.11**	**−0.30**	**0.07**	**< 0.001**	**−0.10**
Marines	0.03	0.07	0.73	0.01	−0.01	0.08	0.94	−0.002	0.04	0.07	0.63	0.01
Navy	0.08	0.07	0.32	0.03	−0.12	0.08	0.18	−0.04	−0.05	0.07	0.62	−0.02
NGR	**−0.20**	**0.09**	**0.031**	**−0.08**	**−0.40**	**0.09**	**< 0.001**	**−0.14**	**−0.40**	**0.08**	**< 0.001**	**−0.15**
Black	**0.33**	**0.08**	**0.001**	**0.08**	**0.36**	**0.10**	**< 0.001**	**0.08**	0.14	0.09	0.21	0.03
Hispanic	0.08	0.07	0.35	0.03	**0.22**	**0.08**	**0.009**	**0.07**	0.11	0.07	0.21	0.04
APINH	**0.47**	**0.14**	**0.003**	**0.07**	0.13	0.17	0.50	0.02	0.13	0.15	0.54	0.02
Multiracial/ethnic	**0.35**	**0.10**	**0.001**	**0.08**	**0.41**	**0.12**	**0.001**	**0.08**	**0.27**	**0.11**	**0.034**	**0.06**
Other race/ethnicity	**0.57**	**0.22**	**0.024**	**0.04**	0.26	0.30	0.44	0.02	0.11	0.28	0.70	0.01
Service support	−0.01	0.07	0.88	−0.01	−0.11	0.08	0.18	−0.05	0.04	0.07	0.63	0.02
Combat support	0.03	0.07	0.73	0.02	0.07	0.07	0.44	0.03	0.10	0.06	0.21	0.04

*Note:* Reference group for gender is female. Reference group for service component is Army. Reference group for race/ethnicity is White. Reference group for military occupation is combat arms. The top half of table displays results for the COVID‐19 pandemic stress outcomes regressed on the mental health predictors and covariates. The bottom half of the table displays results for the mental health predictors regressed on the covariates. Statistically significant results are bolded.

Abbreviations: APINH = asian, pacific islander, native hawaiian; *Β* = standardized regression coefficient; *B* = unstandardized regression coefficient; NGR = national guard/reserve; *p* = *p*‐value (corrected for multiple testing using the false discovery rate method); PTSD = post‐traumatic stress disorder; SE = standard error.

MI symptoms were found to be a statistically significant predictor of COVID‐related work (*B* = 0.11, *p* = 0.011) and financial (*B* = 0.12, *p* = 0.009) stress. PTSD symptoms were not found to be a statistically significant predictor of any COVID‐related stress outcomes. Depression and anxiety symptoms were found to be a statistically significant predictor of all COVID‐related stress domains: social (*B* = 0.28, *p* < 0.001), work (*B* = 0.21, *p* = 0.006), financial (*B* = 0.18, *p* = 0.027), and health (*B* = 0.33, *p* < 0.001).

#### 3.2.1 Sensitivity Analyses

When the structural model was re‐estimated using listwise deletion (*n* = 2451), the SRMR (0.035) and RMSEA (0.043, 90% CI [0.042, 0.044]) indices were comparable to those obtained using multiple imputation. The *χ* ^*2*^ statistic was smaller (*χ* ^*2*^[804] = 4464.51, *p* < 0.001), and the CFI statistic was larger (0.957). The results for MI and PTSD were extremely similar to those found using multiple imputation (see Supporting Information S1: Table 1). Results were also highly similar for anxiety and depression symptoms with the exception of COVID‐related financial stress in which the association was statistically nonsignificant (*B* = 0.17, *p* = 0.06).

When the structural model was re‐estimated using MLR and FIML (*n* = 3180), the SRMR (0.042) was higher than that obtained with WLSMV and both missing data approaches, though the SRMR was still well within acceptable limits. The RMSEA (0.045, 90% CI [0.044, 0.046]) was comparable to the values obtained with WLSMV and both missing data approaches. The *χ* ^*2*^ (*χ* ^*2*^[804] = 4588.82, *p* < 0.001) was closer to the value obtained with WLSMV and listwise deletion compared to multiple imputation, and the CFI (0.922) was closer to the value obtained with WLSMV and multiple imputation compared to listwise deletion. The results for moral injury, PTSD, and depression and anxiety symptoms were highly consistent with those found using WLSMV and multiple imputation (see Supporting Information S1: Table 2).

## 4 Discussion

This study examined the extent to which MI symptoms experienced before the COVID pandemic predicted the degree of stress experienced across various life domains during the pandemic. We hypothesized that pre‐COVID MI symptomology would be associated with veterans' levels of social, work, financial, and health stress during the COVID pandemic. This prediction was partially supported. MI was a statistically significant predictor of COVID‐related work stress and financial stress. It was not a predictor of social or health stress. That MI would predict work and financial stress should not be surprising. The COVID pandemic led to enormous economic, employment, and financial upheaval. For example, unemployment rose, businesses temporarily or permanently closed, and many people had difficulties managing household and other financial responsibilities (Ali and Mahboob 2020; Barlow and Vodenska 2021; Bianchi et al. 2023). Previous studies have hypothesized that certain PMIEs can disrupt an individual's worldview, which may lead to several undesirable psychological and emotional experiences, such as cognitive confusion, uncertainty, apathy, disillusionment, and despair (Fleming and Smigelsky 2024). Thus, it is possible that veterans with higher levels of MI symptomology pre‐COVID may have experienced a greater sense of cognitive confusion and uncertainty during the COVID pandemic, which, in turn, may have resulted in them feeling increased work and financial stress. Cognitive confusion and negative emotions make it much more difficult to concentrate and focus both at work and at home (Gaillard 2018).

It is not clear, however, why MI was not a unique predictor of COVID‐related health or social‐related stress or why PTSD was not a unique predictor of any domains of COVID‐related stress. Perhaps, the most parsimonious explanation is problematic measurement. The latent factors for depression, anxiety, and PTSD in the measurement model were correlated at above 0.80. The correlation between depression and anxiety exceeded a commonly recommended threshold of 0.85 (Brown 2006) and were combined, but, since PTSD did not correlate with depression or anxiety above the 0.85 threshold, the authors decided, on conceptual grounds, to leave PTSD in the model as a unique latent factor. Other threshold cutoffs have been proposed for determining when latent factors are too highly correlated, for example, *r* = 0.70 (Shao et al. 2022). Thus, the SEM analyses may have encountered issues in accurately estimating the unique effects of highly correlated predictor variables (e.g., biased parameter estimates, inflated standard errors [Grewal et al. 2004]). Another possible explanation is that the model parameters were estimated without issue, and when controlling for the other variables in the model, MI and PTSD did not explain variability in health or social COVID‐related stress. For instance, the MI measure used in this study includes three items that ask about self‐directed MI. Self‐directed MI tends to be associated with self‐hate and beliefs that one is tarnished and irredeemable. People with these feelings often believe they should be alone and isolated (Seagraves 2025) and, hence, may not have been distressed by social isolation during the pandemic. Further, the lack of association between MI and health‐related stress may lie in the fact that MI is largely a cognitive and emotional phenomenon that impacts one's mental health (Griffin et al. 2019; Hall et al. 2022) but not necessarily his or her physical health.

Similar arguments could be made about why PTSD symptoms were not a predictor of any domains of COVID‐related stress above and beyond the other covariates in the model, especially social stress. Given that PTSD is largely a disorder of avoidance (Sheynin et al. 2017), veterans with PTSD symptoms were, perhaps, in some way comforted by the social isolation that resulted from the COVID pandemic. Indeed, PTSD had a low correlation with social stress (*r* = 0.17) in the measurement model. Further research examining these assertions about MI and PTSD symptomology insulating veterans from COVID‐related stress due to perceptions of social isolation as penance or relief is needed. Social isolation is a serious issue affecting veterans, especially older veterans (G. Wilson et al. 2018) and given social restriction mandates put in place in various communities during the COVID pandemic, increased social isolation was identified early on as a pernicious effect of the pandemic (Hwang et al. 2020). While studies examining social isolation, and the closely related concept of loneliness, in veterans during the COVID pandemic generally seem to find that, overall, veterans were resilient (McGuire et al. 2023; Na et al. 2022), there were some indications that social isolation and loneliness increased or persisted for some veterans (Kalvesmaki et al. 2022; S. Li et al. 2023; Na et al. 2022; Purcell et al. 2021). One study found that veterans' reports of loneliness, as assessed by the UCLA loneliness scale short form, were greater than civilians during the COVID pandemic (Umucu et al. 2022).Pre‐COVID psychiatric symptoms have been shown to be associated with veterans' perceptions of social isolation and loneliness during the pandemic (Na et al. 2022), though no pandemic‐focused studies that include MI or examine veterans' rationale for social isolation appear to exist. Quantitative investigations employing mediation and moderation analyses and qualitative studies exploring the depth of veterans' lived experiences are necessary to thoroughly understand the interplay among MI and PTSD symptomology, social isolation and loneliness, and COVID‐related stress.

Another reason for the lack of significant findings could be due to the normative nature of the sample. The participants in this study were not diagnosed with PTSD. Perhaps, results with a clinical sample may have yielded stronger associations between PTSD and the COVID stress‐related outcomes. Studies have found that individuals with psychiatric diagnoses bore a significant stress burden during the pandemic (Belz et al. 2022; S. Li and Zhang 2020). Further, while MI is not yet established as a diagnosable condition (Litz 2025; Litz and Walker 2025) and can be particularly challenging to measure due to conceptual issues and measurement scale limitations, which includes the scale used in the present study (Litz and Walker 2025), there was no indication that study participants were experiencing high levels of MI‐related distress based on the observed means of the MISS‐M‐SF4 items used in the analyses (*M*_*range*_: 1.80–3.71 on a 10‐point scale). Future studies should consider the effects of other highly isolating and stressful events on veterans who have a diagnosis of PTSD and may also be experiencing severe MI, such as physical impairment leading to less socialization; loss of socially supportive loved ones through divorce, death or other reasons; remote employment while disconnected to one's community; social media overuse; or unemployment.

Symptoms of depression and anxiety were positively associated with each type of COVID‐related stress. People with depression and anxiety are susceptible to feeling ‘stressed out’ and overwhelmed in the face of challenging situations (Bergdahl and Bergdahl 2002; Phillips et al. 2015). Individuals with mental health problems, such as depression and anxiety, tend to not use constructive coping strategies (Aldao et al. 2010). For example, studies have found that individuals who have depression are more likely to use avoidance and denial coping compared to those who do not have mental health problems (Orzechowska et al. 2013) and are less likely to use problem‐focused coping (Clarke and Goosen 2009). Similarly, individuals who have anxiety use less adaptive coping strategies than those who do not have anxiety (Thomasson and Psouni 2010). Future studies should examine the coping strategies used by veterans who have depression and anxiety in the face of difficult life events. It may be, for instance, that veterans experiencing depression and anxiety symptoms who have also encountered PMIEs may utilize different coping mechanisms for future stressors depending on the nature of the PMIE (i.e., self‐vs. other‐directed). In this way, PMIEs could operate as a moderator. While quantitative studies would be able to examine moderating effects of PMIEs on coping strategies, qualitative studies should also be pursued to provide greater nuance to the complex ways that depression, anxiety, moral injury, and coping are undoubtedly connected.

This study's findings should be interpreted considering its limitations. First, all data come from self‐report measures, and, as such, are susceptible to several forms of bias, such as recall, social desirability, and item order effects. Second, the MI measure used in this study does not capture specific subtypes of MI (e.g., self‐ and other‐directed MI). Rather, the measure assesses a general MI symptoms factor, and as such, no inferences about the effects of self‐ and other‐directed MI on COVID‐related stress can be made. Third, as previously mentioned, several of the mental health variables were highly correlated. This may indicate issues with the discriminant validity of the scales when used with this sample of post 9/11 U.S. veterans. Highly correlated predictor variables can sometimes make it challenging for statistical models to produce accurate parameter estimates and standard errors, which, in turn, can lead to difficulties in interpreting results. Fourth, regional differences in the restrictions imposed to combat the spread of COVID‐19 likely impacted individuals' mental health (Ferwana and Varshney 2024); however, the authors did not have access to participants' location data and were unable to control for potential geographical differences in the analyses. Fifth, missing data were accounted for via multiple imputation, which works well when the data are missing at random (MAR). Unfortunately, MAR is an untestable assumption (Enders 2010), and if this assumption is violated, model parameters may be biased. Two sensitivity analyses were conducted, and the results of these sensitivity analyses were highly similar to the results obtained through multiple imputation. Thus, it is plausible that the MAR assumption was not violated. Sixth, though TVMI and VETS are longitudinal studies, only two waves of data were able to be used in this study. The flexibility of statistical models for examining longitudinal associations is limited when there are only two waves of data. Further, only being able to use two waves of data precluded the investigation of mental health as a mediator of the relationship between MI symptomology and COVID‐related stress. Given the associations among MI and mental health outcomes documented by previous research (Griffin et al. 2019; Hall et al. 2022), it is entirely plausible that MI impacts COVID‐related stress through mental health symptomology. Of course, as causality has not yet been clearly established, it is also possible that MI serves as a mediator of the associations between mental health outcomes and COVID‐related stress. Exploring mediational pathways would be an important focus for future research. Finally, while the initial sample that participated in TVMI was representative of post‐9/11 veterans who had transitioned out of the military between August and November of 2016, only a subset of TVMI veterans participated in VETS, which likely limits the generalizability of the findings. Given the study's sample, it is unclear the extent to which these findings would generalize beyond post‐9/11 samples.

The limitations of this study notwithstanding, the findings underscore the nuances in how veterans' pre‐COVID pandemic psychological symptoms, including MI, affected the stress they experienced in various life domains during the COVID pandemic. The study's findings are bolstered by the large sample size, the longitudinal design, and the results of the main analyses converging with the results of the sensitivity analyses. Hopefully, the findings of this study can inform future research focused on the well‐being of veterans within the context of highly stressful life events.

## Funding

The Veterans Metrics Initiative (TVMI) research was managed by the Henry M. Jackson Foundation for the Advancement of Military Medicine Inc. (HJF), and it was collaboratively sponsored by the Bob Woodruff Foundation, Health Net Federal Services, HJF, Lockheed Martin Corporation, Marge and Philip Odeen, May and Stanley Smith Charitable Trust, National Endowment for the Humanities, Northrop Grumman, Prudential, Robert R. McCormick Foundation, Rumsfeld Foundation, Schultz Family Foundation, The Heinz Endowments, U.S. Department of Veterans Affairs Health Services Research and Development Service, Walmart Foundation, and Wounded Warrior Project Inc. Support for the Veterans Engaging in Transition Studies (VETS) Wave 7 survey was provided by The Pew Charitable Trusts. The views expressed are those of the author(s) and do not necessarily reflect the views of The Pew Charitable Trusts. The lead Pennsylvania State University Principal Investigator and many of the authors are employed at the Clearinghouse for Military Family Readiness at Penn State. This academic unit is the result of a partnership funded by the Department of Defence as acknowledged by the following statement. The Clearinghouse for Military Family Readiness at Penn State is the result of a partnership funded by the Department of Defence between the Office of the Deputy Assistant Secretary of Defence for Military Community and Family Policy and the USDA's National Institute of Food and Agriculture through a cooperative agreement with the Pennsylvania State University. This work leverages funds by the USDA's National Institute of Food and Agriculture and Hatch Appropriations.

## Conflicts of Interest

The authors declare no conflicts of interest.

## Disclaimer

The views expressed in this article are those of the authors and not an official position of any institution or funder. The investigators followed the policies governing the protection of human subjects as prescribed by ICF International Inc.’s Institutional Review Board and the Pennsylvania State University's Institutional Review Board.

## Supporting information


Supporting Information S1

## Data Availability

TVMI data have been made publicly available at the Inter‐University Consortium for Political and Social Research and can be accessed at https://www.icpsr.umich.edu/ICPSR/studies/38051/summary. VETS data have not yet been made publicly available.
