# Supplementary material for: Moral Injury and COVID‐Related Stress Among Post‐9/11 Veterans: Examining Longitudinal Associations
Source: Stress Health. 2025 Nov 10;41(6):e70121. doi: 10.1002/smi.70121 (PMC12603346; doi:10.1002/smi.70121)
Supplement: Supplementary file 1 — Supporting Information S1 [file SMI-41-e70121-s001.docx]

**Supplemental Table 1**

*Structural Model Regression Results Using Listwise Deletion*

|  | COVID Social Stress | | | | COVID Work Stress | | | | COVID Financial Stress | | | | COVID Health Stress | | | |
| --- | --- | --- | --- | --- | --- | --- | --- | --- | --- | --- | --- | --- | --- | --- | --- | --- |
| Mental Health Predictors & Covariates | *B* | *SE* | *p* | *β* | *B* | *SE* | *p* | *β* | *B* | *SE* | *p* | *β* | *B* | *SE* | *p* | *β* |
| Moral Injury | -.03 | .03 | .57 | -.03 | **.12** | **.04** | **.005** | **.12** | **.12** | **.04** | **.014** | **.11** | .02 | .04 | .76 | .02 |
| PTSD | -.06 | .06 | .48 | -.07 | -.05 | .07 | .73 | -.06 | .15 | .07 | .16 | .15 | .03 | .07 | .76 | .03 |
| Depression & Anxiety | **.30** | **.06** | **<.001** | **.30** | **.20** | **.07** | **.007** | **.20** | .17 | .07 | .06 | .16 | **.35** | **.07** | **<.001** | **.34** |
| Combat Exposure | -.01 | .04 | .84 | -.01 | .01 | .04 | .95 | .01 | -.06 | .04 | .41 | -.05 | -.04 | .04 | .40 | -.04 |
| Gender | **-.32** | **.07** | **<.001** | **-.11** | .02 | .07 | .95 | .01 | -.02 | .07 | .92 | -.01 | **-.40** | **.07** | **<.001** | **-.13** |
| Paygrade | **.09** | **.02** | **<.001** | **.14** | **.06** | **.02** | **.002** | **.09** | **-.15** | **.02** | **<.001** | **-.22** | **.08** | **.02** | **<.001** | **.11** |
| Air Force | **.20** | **.07** | **.021** | **.07** | **.22** | **.08** | **.007** | **.07** | .07 | .08 | .68 | .02 | .05 | .08 | .70 | .02 |
| Marines | .08 | .07 | .48 | .03 | -.03 | .08 | .95 | -.01 | .08 | .08 | .68 | .03 | -.10 | .08 | .31 | -.04 |
| Navy | .10 | .07 | .48 | .04 | .01 | .08 | .95 | .002 | .01 | .08 | .92 | .004 | -.03 | .08 | .76 | -.01 |
| NGR | .06 | .08 | .57 | .02 | **.30** | **.09** | **.003** | **.11** | -.01 | .09 | .92 | -.003 | .001 | .09 | .99 | .0004 |
| Black | -.10 | .09 | .48 | -.03 | **.33** | **.10** | **.003** | **.08** | .16 | .10 | .40 | .04 | **.34** | **.10** | **.002** | **.08** |
| Hispanic | -.07 | .08 | .48 | -.02 | **.35** | **.08** | **<.001** | **.11** | .07 | .08 | .68 | .02 | **.25** | **.08** | **.005** | **.08** |
| APINH | .36 | .16 | .08 | .05 | **.54** | **.17** | **.005** | **.08** | .04 | .18 | .92 | .01 | **.42** | **.15** | **.014** | **.06** |
| Multiracial/ethnic | .06 | .10 | .60 | .01 | **.45** | **.12** | **.001** | **.10** | -.03 | .11 | .92 | -.01 | .22 | .11 | .10 | .05 |
| Other Race/Ethnicity | -.40 | .34 | .48 | -.03 | -.07 | .33 | .95 | -.01 | .03 | .28 | .92 | .002 | -.13 | .32 | .76 | -.01 |
| Service Support | .07 | .07 | .48 | .03 | -.01 | .08 | .95 | -.01 | -.07 | .08 | .68 | -.03 | **.22** | **.07** | **.008** | **.09** |
| Combat Support | .02 | .07 | .78 | .01 | .14 | .07 | .09 | .07 | -.02 | .08 | .92 | -.01 | **.22** | **.07** | **.006** | **.10** |
|  |  |  |  |  |  |  |  |  |  |  |  |  |  |  |  |  |
|  | Moral Injury | | | | PTSD | | | | Depression & Anxiety | | | |  |  |  |  |
| Covariates | *B* | *SE* | *p* | *β* | *B* | *SE* | *p* | *β* | *B* | *SE* | *p* | *β* |  |  |  |  |
| Combat Exposure | **.17** | **.03** | **<.001** | **.17** | **.46** | **.04** | **<.001** | **.40** | **.25** | **.03** | **<.001** | **.23** |  |  |  |  |
| Gender | **-.26** | **.07** | **<.001** | **-.09** | **-.37** | **.08** | **<.001** | **-.12** | **-.32** | **.07** | **<.001** | **-.11** |  |  |  |  |
| Paygrade | **-.07** | **.02** | **<.001** | **-.11** | **-.06** | **.02** | **.004** | **-.08** | **-.09** | **.02** | **<.001** | **-.13** |  |  |  |  |
| Air Force | **-.16** | **.07** | **.047** | **-.05** | **-.41** | **.08** | **<.001** | **-.12** | **-.33** | **.07** | **<.001** | **-.11** |  |  |  |  |
| Marines | .07 | .08 | .49 | .03 | .03 | .08 | .69 | .01 | .08 | .08 | .44 | .03 |  |  |  |  |
| Navy | .06 | .08 | .53 | .02 | -.12 | .08 | .21 | -.04 | -.04 | .08 | .64 | -.01 |  |  |  |  |
| NGR | **-.20** | **.09** | **.047** | **-.07** | **-.40** | **.09** | **<.001** | **-.13** | **-.41** | **.08** | **<.001** | **-.15** |  |  |  |  |
| Black | **.30** | **.09** | **.002** | **.08** | **.34** | **.10** | **.003** | **.08** | .06 | .09 | .59 | .02 |  |  |  |  |
| Hispanic | .05 | .08 | .64 | .02 | .18 | .09 | .078 | .05 | .10 | .08 | .36 | .03 |  |  |  |  |
| APINH | **.45** | **.15** | **.006** | **.07** | .09 | .19 | .66 | .01 | .13 | .16 | .59 | .02 |  |  |  |  |
| Multiracial/ethnic | **.37** | **.10** | **.001** | **.08** | **.43** | **.13** | **.003** | **.09** | .27 | .12 | .06 | .06 |  |  |  |  |
| Other Race/Ethnicity | **.60** | **.24** | **.024** | **.04** | .39 | .34 | .32 | .03 | .16 | .34 | .64 | .01 |  |  |  |  |
| Service Support | -.04 | .08 | .67 | -.02 | -.12 | .08 | .21 | -.05 | .05 | .07 | .59 | .02 |  |  |  |  |
| Combat Support | .03 | .07 | .67 | .02 | .08 | .08 | .33 | .04 | .12 | .07 | .18 | .05 |  |  |  |  |

*Note.* PTSD = Post-traumatic stress disorder. NGR = National Guard/Reserve. APINH = Asian, Pacific Islander, Native Hawaiian. Reference group for gender is female. Reference group for service component is Army. Reference group for race/ethnicity is White. Reference group for military occupation is combat arms. The top half of table displays results for the COVID-19 pandemic stress outcomes regressed on the mental health predictors and covariates. The bottom half of the table displays results for the mental health predictors regressed on the covariates. *B* = Unstandardized regression coefficient. *SE* = Standard error. *p* = *p*-value (corrected for multiple testing using the false discovery rate method). *Β* = Standardized regression coefficient. Statistically significant results are bolded.

**Supplemental Table 2**

*Structural Model Regression Results Using MLR and FIML*

|  | COVID Social Stress | | | | COVID Work Stress | | | | COVID Financial Stress | | | | COVID Health Stress | | | |
| --- | --- | --- | --- | --- | --- | --- | --- | --- | --- | --- | --- | --- | --- | --- | --- | --- |
| Mental Health Predictors & Covariates | *B* | *SE* | *p* | *β* | *B* | *SE* | *p* | *β* | *B* | *SE* | *p* | *β* | *B* | *SE* | *p* | *β* |
| Moral Injury | -.04 | .04 | .55 | -.04 | **.12** | **.05** | **.027** | **.12** | **.17** | **.05** | **.008** | **.16** | -.01 | .05 | .85 | -.01 |
| PTSD | -.04 | .05 | .58 | -.04 | -.06 | .06 | .39 | -.07 | .08 | .06 | .53 | .07 | .02 | .06 | .75 | .02 |
| Depression & Anxiety | **.27** | **.05** | **<.001** | **.28** | **.18** | **.06** | **.008** | **.18** | **.17** | **.06** | **.02** | **.16** | **.35** | **.06** | **<.001** | **.33** |
| Combat Exposure | .01 | .03 | .83 | .01 | .01 | .03 | .98 | .01 | -.02 | .03 | .71 | -.02 | -.01 | .03 | .75 | -.01 |
| Gender | **-.38** | **.06** | **<.001** | **-.13** | -.01 | .07 | .98 | -.004 | -.04 | .06 | .71 | -.01 | **-.42** | **.06** | **<.001** | **-.14** |
| Paygrade | **.08** | **.01** | **<.001** | **.11** | **.04** | **.02** | **.017** | **.06** | **-.13** | **.01** | **<.001** | **-.18** | **.06** | **.01** | **<.001** | **.08** |
| Air Force | **.25** | **.06** | **<.001** | **.08** | **.16** | **.07** | **.042** | **.05** | .05 | .07 | .71 | .01 | .06 | .07 | .55 | .02 |
| Marines | .09 | .07 | .38 | .03 | -.08 | .07 | .39 | -.03 | .04 | .07 | .71 | .02 | -.14 | .07 | .09 | -.05 |
| Navy | .13 | .07 | .14 | .05 | .001 | .07 | .99 | .0002 | .02 | .07 | .81 | .01 | -.03 | .07 | .75 | -.01 |
| NGR | .08 | .07 | .43 | .03 | **.18** | **.07** | **.027** | **.07** | -.04 | .07 | .71 | -.01 | -.07 | .07 | .53 | -.02 |
| Black | -.06 | .08 | .61 | -.01 | **.30** | **.10** | **.007** | **.07** | .23 | .10 | .07 | .05 | **.33** | **.08** | **<.001** | **.08** |
| Hispanic | -.01 | .07 | .91 | -.004 | **.30** | **.08** | **.001** | **.10** | .15 | .07 | .10 | .05 | **.30** | **.07** | **<.001** | **.09** |
| APINH | **.43** | **.13** | **.001** | **.06** | **.61** | **.17** | **.002** | **.09** | .11 | .14 | .71 | .02 | **.56** | **.13** | **<.001** | **.08** |
| Multiracial/ethnic | .01 | .10 | .91 | .002 | **.35** | **.11** | **.007** | **.08** | .04 | .10 | .81 | .01 | .24 | .11 | .06 | .05 |
| Other Race/Ethnicity | -.40 | .23 | .21 | -.03 | -.01 | .24 | .99 | -.001 | .08 | .26 | .81 | .01 | -.17 | .30 | .74 | -.01 |
| Service Support | .06 | .06 | .55 | .03 | .04 | .07 | .71 | .02 | -.05 | .07 | .71 | -.02 | **.19** | **.07** | **.012** | **.08** |
| Combat Support | .04 | .06 | .61 | .02 | **.18** | **.07** | **.017** | **.08** | -.01 | .06 | .84 | -.01 | **.19** | **.06** | **.008** | **.08** |
|  |  |  |  |  |  |  |  |  |  |  |  |  |  |  |  |  |
|  | Moral Injury | | | | PTSD | | | | Depression & Anxiety | | | |  |  |  |  |
| Covariates | *B* | *SE* | *p* | *β* | *B* | *SE* | *p* | *β* | *B* | *SE* | *p* | *β* |  |  |  |  |
| Combat Exposure | **.14** | **.03** | **<.001** | **.13** | **.34** | **.03** | **<.001** | **.31** | **.20** | **.03** | **<.001** | **.19** |  |  |  |  |
| Gender | **-.23** | **.07** | **.004** | **-.08** | **-.40** | **.07** | **<.001** | **-.13** | **-.35** | **.06** | **<.001** | **-.12** |  |  |  |  |
| Paygrade | **-.09** | **.01** | **<.001** | **-.14** | **-.09** | **.01** | **<.001** | **-.13** | **-.10** | **.01** | **<.001** | **-.15** |  |  |  |  |
| Air Force | -.10 | .06 | .17 | -.03 | -.12 | .06 | .08 | -.04 | **-.16** | **.07** | **.036** | **-.05** |  |  |  |  |
| Marines | .08 | .08 | .33 | .03 | .13 | .07 | .12 | .04 | .13 | .07 | .11 | .05 |  |  |  |  |
| Navy | .14 | .08 | .15 | .05 | .12 | .07 | .12 | .04 | .09 | .07 | .21 | .03 |  |  |  |  |
| NGR | -.10 | .08 | .26 | -.04 | -.12 | .07 | .12 | -.04 | **-.22** | **.07** | **.008** | **-.08** |  |  |  |  |
| Black | .25 | .11 | .06 | .06 | **.38** | **.11** | **.001** | **.09** | .16 | .09 | .13 | .04 |  |  |  |  |
| Hispanic | .06 | .07 | .42 | .02 | **.20** | **.08** | **.015** | **.06** | .12 | .07 | .13 | .04 |  |  |  |  |
| APINH | .41 | .20 | .09 | .06 | .12 | .15 | .44 | .02 | .15 | .16 | .38 | .02 |  |  |  |  |
| Multiracial/ethnic | **.35** | **.13** | **.021** | **.08** | **.34** | **.12** | **.007** | **.07** | **.24** | **.11** | **.042** | **.05** |  |  |  |  |
| Other Race/Ethnicity | .51 | .36 | .24 | .04 | .24 | .27 | .39 | .02 | .01 | .19 | .94 | .001 |  |  |  |  |
| Service Support | .07 | .07 | .33 | .03 | .11 | .06 | .11 | .05 | **.15** | **.06** | **.036** | **.07** |  |  |  |  |
| Combat Support | .09 | .07 | .26 | .04 | **.20** | **.06** | **.003** | **.09** | **.17** | **.06** | **.014** | **.08** |  |  |  |  |

*Note.* MLR = Robust maximum likelihood. FIML = Full information maximum likelihood. PTSD = Post-traumatic stress disorder. NGR = National Guard/Reserve. APINH = Asian, Pacific Islander, Native Hawaiian. Reference group for service component is Army. Reference group for gender is female. Reference group for race/ethnicity is White. Reference group for military occupation is combat arms. The top half of table displays results for the COVID-19 pandemic stress outcomes regressed on the mental health predictors and covariates. The bottom half of the table displays results for the mental health predictors regressed on the covariates. *B* = Unstandardized regression coefficient. *SE* = Standard error. *p* = *p*-value (corrected for multiple testing using the false discovery rate method). *Β* = Standardized regression coefficient. Statistically significant results are bolded.
